# Supplementary material for: On demand shape memory polymer via light regulated topological defects in a dynamic covalent network
Source: Nat Commun. 2020 Aug 26;11:4257. doi: 10.1038/s41467-020-18116-1 (PMC7450050; doi:10.1038/s41467-020-18116-1)
Supplement: Supplementary file 1 — Supplementary Information [file 41467_2020_18116_MOESM1_ESM.pdf]

Supplementary Information

On Demand Shape Memory Polymer via Light Regulated Topological Defects in a Dynamic Covalent Network

Miao et al.

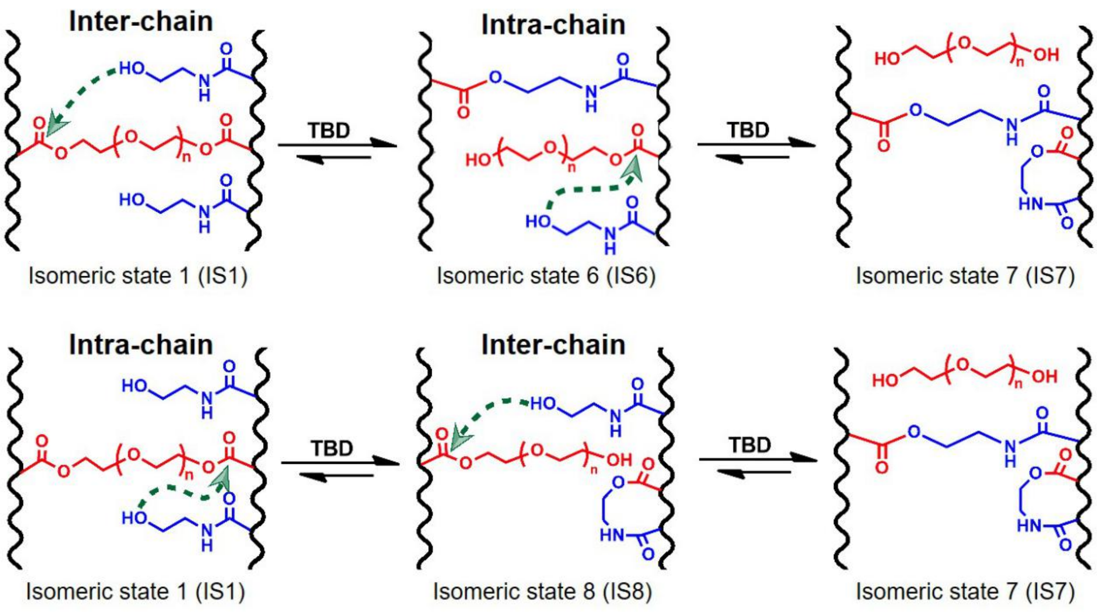

Supplementary Figure 1 Network isomerization via the inter- and intra-chain transesterification in an intermixed fashion. Relative to the original isomeric state (IS1), the number of crosslinking points remain unchanged for IS6 and IS7 while IS8 decreases.

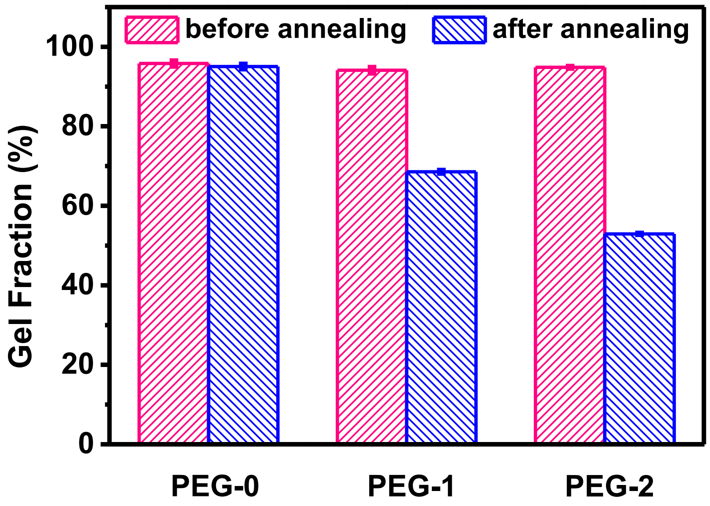

Supplementary Figure 2 Gel fraction of PEG-0, PEG-1 and PEG-2 samples before and after thermal treatment. Error bars represent standard deviations caculated from three specimens.

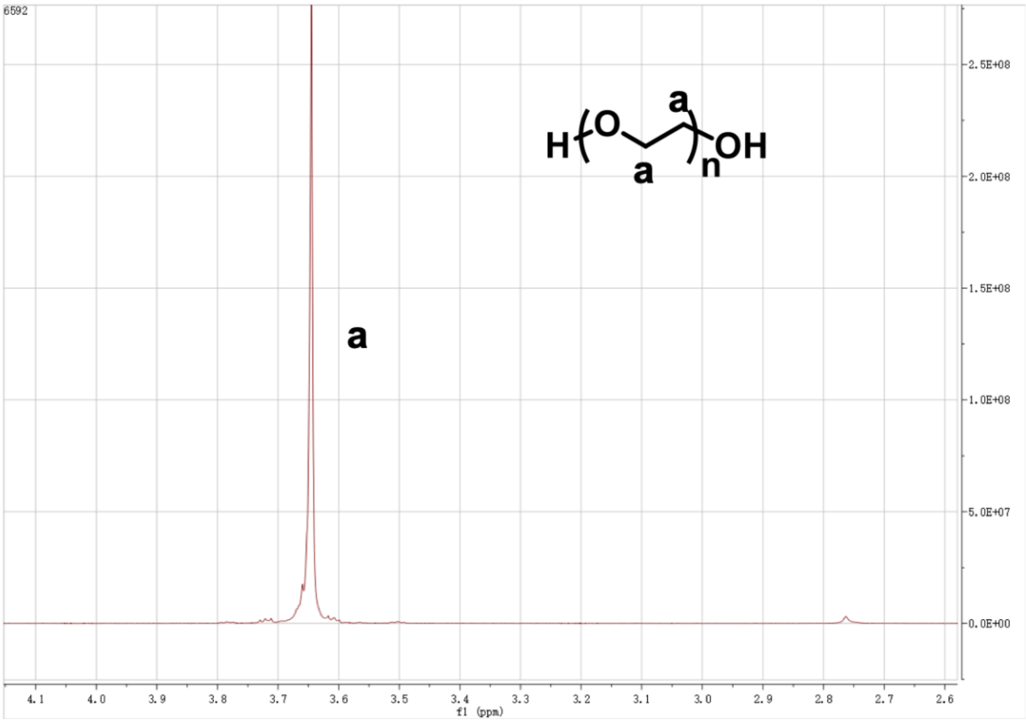

Supplementary Figure 3 ^{1}H NMR spectra of PEG extracted from the thermally annealed PEG-2 samples. (All ^{1}H NMR analysis was recorded with Bruker Avance 500 (500 MHz) using $CDCl_3$ as the solvent)

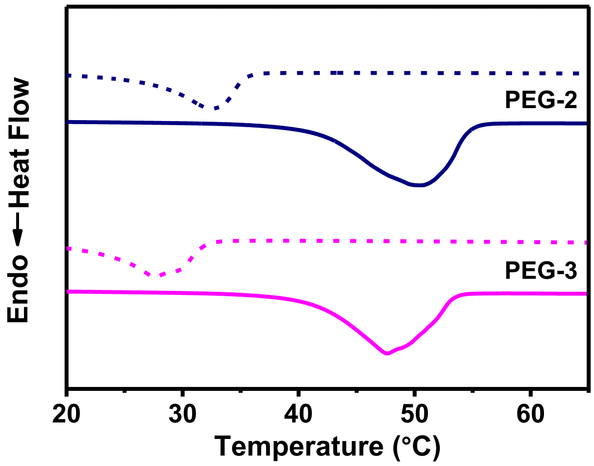

Supplementary Figure 4 DSC curves of PEG-2 and PEG-3 before (dotted lines) and after (solid lines) isomerization.

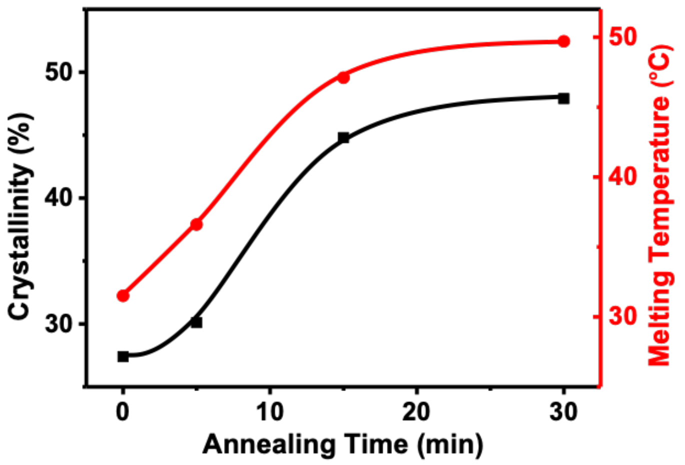

Supplementary Figure 5 Kinetic studies of the PEG-2 isomerization (irradiation time: 300 s). The crystallinity and melting temperatures of the isomerized PEG-2 samples.

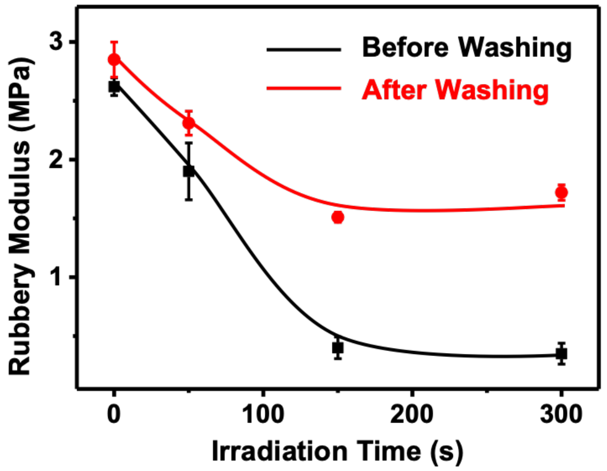

Supplementary Figure 6 Rubbbery moduli (80 °C) of the isomerized (washed) samples upon irradiation of different time. Error bars represent standard deviations caculated from three specimens.

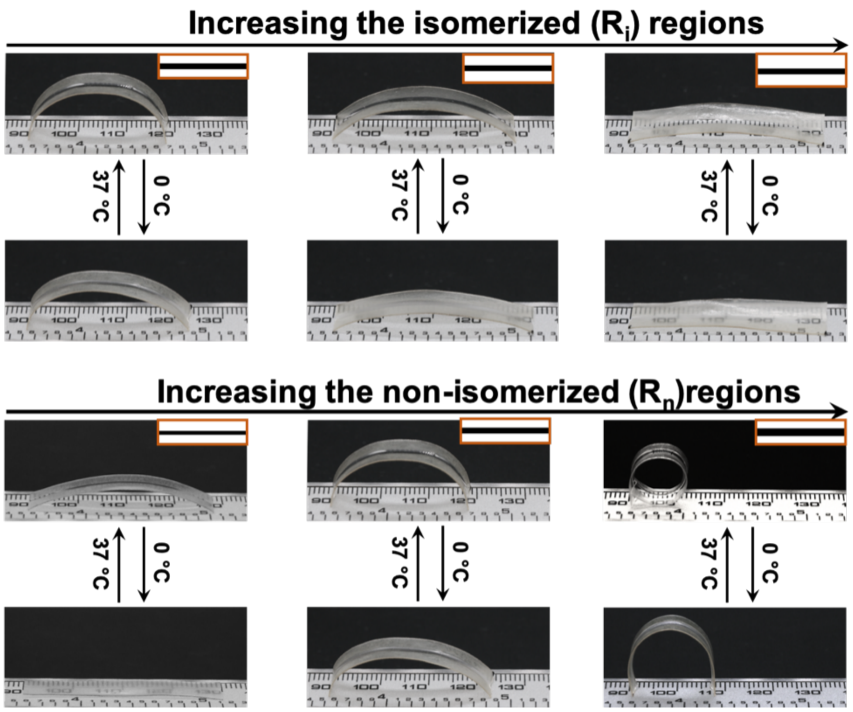

Supplementary Figure 7. Tunable bending curvature of reversible shape memory behavior (the corresponding light patterns shown as the insets, pre-stretching: 30 %).

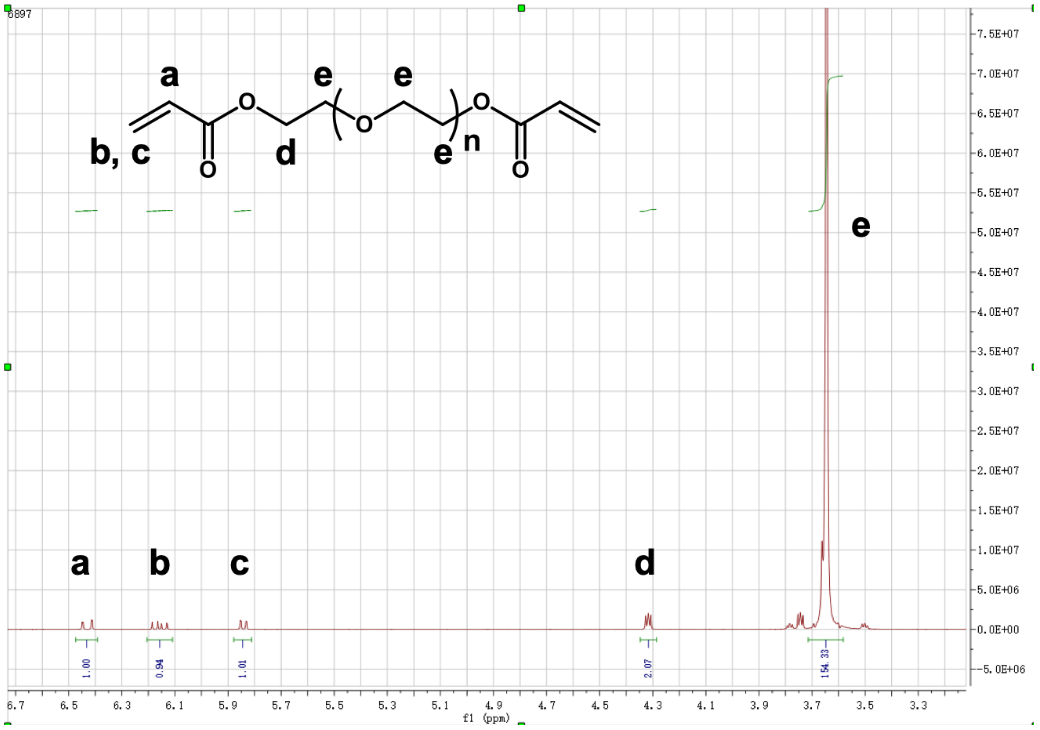

Supplementary Figure 8 ^{1}H NMR spectra of PEGDA.
